# CDK7 as a Potential Exploratory Biomarker for Distinguishing Acute Myocardial

**DOI:** 10.1002/clc.70396

**Published:** 2026-06-24

**Authors:** Awais Mehmood, Romesa Tahir

**Affiliations:** ^1^ Continental Medical College Lahore Pakistan; ^2^ University of Health Sciences Lahore Pakistan

Dear Editor,

We read with interest the article by Ripon et al. regarding the differential expression of DNA damage response (DDR) genes across STEMI and NSTEMI subtypes in a Bangladeshi cohort [1]. This study provides valuable data on South Asian populations, an underrepresented group in cardiovascular genomics. The authors' identification of CDK7 as a subtype‐specific gene elevated in NSTEMI and significantly after clinical adjustment is a notable finding.

However, we must raise a significant methodological concern regarding the exclusive use of GAPDH as the reference gene for qRT‐PCR normalization. This choice has substantial implications for the interpretation of the findings. GAPDH encodes a central glycolytic enzyme, and its expression is well‐documented to fluctuate under the hypoxic and oxidative stress conditions intrinsic to acute myocardial ischemia. As demonstrated by Zhong and Simons [2], GAPDH mRNA levels are significantly upregulated during hypoxia, whereas many other housekeeping genes remain stable.

In the context of this study, this issue is critical because STEMI and NSTEMI differ fundamentally in ischemic severity. STEMI involves complete coronary occlusion and more profound hypoxia, while NSTEMI typically involves partial occlusion with relatively preserved perfusion. If GAPDH expression varies in proportion to the ischemic burden, a biologically plausible scenario, its use as a normalization control introduces a systematic bias. It is possible that the observed upregulation of CDK7 in NSTEMI relative to STEMI reflects altered GAPDH expression in the more severely ischemic STEMI group rather than a true difference in CDK7 transcriptional activity.

The Minimum Information for Publication of Quantitative Real‐Time PCR Experiments (MIQE) [3] guidelines strongly recommend the use of at least two validated reference genes to ensure normalization reliability. Relying on a single, unvalidated reference gene, especially one prone to context‐dependent variability, fails to meet these rigorous standards. Established tools, such as the geNorm algorithm introduced by Vandesompele et al., [4] provide a framework for identifying the most stable reference genes across specific experimental conditions and represent the standard practice for gene expression studies in pathological states.

We recommend that follow‐up studies validate at least two or three reference genes before normalization. Genes such as HPRT1, B2M, and RPL13A have shown more stable expression under ischemic stress. Tools such as geNorm, NormFinder, or BestKeeper make this achievable and are now widely used in published qRT‐PCR work. On a practical note, if raw GAPDH *C*_t_ values are still accessible, a direct comparison between the STEMI and NSTEMI groups would quickly clarify whether GAPDH fluctuation contributed to the CDK7 differences observed.

Without confirming GAPDH stability across groups, it is genuinely difficult to determine whether the CDK7 difference reflects true subtype‐specific biology or a normalization artifact. This is not a minor technical point; the study's central conclusion depends on it. We appreciate the authors' transparency in outlining study limitations and hope this methodological feedback contributes to the continued success of their research.

## Author Contributions

The authors have read and approved the final version of this letter.

## Funding

The authors have nothing to report.

## Ethics Statement

The authors have nothing to report.

## Consent

The authors have nothing to report.

## Conflicts of Interest

The authors declare no conflicts of interest.

## Data Availability

Data sharing is not applicable to this article as no data sets were generated or analyzed during the current study.
